# Multimodal treatment with curative intent in a germline *BRCA2* mutant metastatic ampullary adenocarcinoma: a case report

**DOI:** 10.1186/s12957-023-02976-0

**Published:** 2023-03-31

**Authors:** Gianluca Mauri, Viviana Gori, Giorgio Patelli, Laura Roazzi, Francesco Rizzetto, Luciano De Carlis, Anna Mariani, Ugo Cavallari, Elisabetta Prada, Tiziana Cipani, Maria Costanza Aquilano, Emanuela Bonoldi, Angelo Vanzulli, Salvatore Siena, Andrea Sartore-Bianchi

**Affiliations:** 1 Department of Oncology and Hemato-Oncology, Università degli Studi di Milano, Piazza Ospedale Maggiore, 3, 20162 Milan, Italy; 2 Department of Hematology, Oncology, and Molecular Medicine, Grande Ospedale Metropolitano Niguarda, Milan, Italy; 3 Department of Services, Grande Ospedale Metropolitano Niguarda, Milan, Italy; 4 Department of Surgery and Transplantation, Grande Ospedale Metropolitano Niguarda, Milan, Italy; 5 Università degli Studi di Milano-Bicocca, Milan, Italy; 6 Department of Laboratory Medicine, Grande Ospedale Metropolitano Niguarda, Milan, Italy

**Keywords:** Case report, Surgical metastasectomy, BRCA, Ampullary cancer, Platinum toxicity and sensitivity

## Abstract

**Background:**

Cancers of the Vater ampulla (ampullary cancers, ACs) account for less than 1% of all gastrointestinal tumors. ACs are usually diagnosed at advanced stage, with poor prognosis and limited therapeutic options. *BRCA2* mutations are identified in up to 14% of ACs and, differently from other tumor types, therapeutic implications remain to be defined. Here, we report a clinical case of a metastatic AC patient in which the identification of a *BRCA2* germline mutation drove a personalized multimodal approach with curative-intent.

**Case presentation:**

A 42-year-old woman diagnosed with stage IV *BRCA2* germline mutant AC underwent platinum-based first line treatment achieving major tumor response but also life-threatening toxicity. Based on this, as well as on molecular findings and expected low impact of available systemic treatment options, the patient underwent radical complete surgical resection of both primary tumor and metastatic lesions. Following an isolated retroperitoneal nodal recurrence, given the expected enhanced sensitivity to radiotherapy in *BRCA2* mutant cancers, the patient underwent imaging-guided radiotherapy leading to long-lasting complete tumor remission. After more than 2 years, the disease remains radiologically and biochemically undetectable. The patient accessed a dedicated screening program for *BRCA2* germline mutation carriers and underwent prophylactic bilateral oophorectomy.

**Conclusions:**

Even considering the intrinsic limitations of a single clinical report, we suggest that the finding of *BRCA* germline mutations in ACs should be taken into consideration, together with other clinical variables, given their potential association with remarkable response to cytotoxic chemotherapy that might be burdened with enhanced toxicity. Accordingly, *BRCA1/2* mutations might offer the opportunity of personalizing treatment beyond PARP inhibitors up to the choice of a multimodal approach with curative-intent.

## Introduction

Cancers of the Vater ampulla (ampullary cancers, ACs) are rare malignancies accounting for less than 1% of all gastrointestinal tumors [1, 2]. The prognosis is poor, and less than 20% of patients are alive at 5 years after the initial diagnosis [3]. Particularly, in stage IV disease, the median overall survival ranges between 15 and 20 months [2–4]. Overall, nearly all ACs are adenocarcinoma and are classified into three different histologic subtypes according to the differentiation, namely pancreatobiliary, intestinal, and mixed — the former with more aggressive behavior sharing similarities with pancreatic and bile duct cancers [5, 6]. *TP53* mutations (41–53%) and *KRAS* mutations (40%) are among the most common alterations found in ACs, while *ERBB2/3* alterations and microsatellite instability (MSI) are rarer but may represent potential therapeutic targets in the metastatic disease [7–9]. Beyond them, pathogenic germline variations in *BRCA2*, *ATM*, *APC*, *MUTYH*, and *RAD50* have been reported to occur in gastrointestinal cancers and up to 18% in AC patients [10]. In particular, *BRCA2* mutations have been reported to occur in around 14% of ACs [11, 12]. However, differently from other cancers [13–15], the therapeutic implications of this molecular finding in AC patients remain to be assessed.

Pancreaticoduodenectomy with lymphadenectomy is the current gold standard treatment for early stages, even though relapses occur in more than half of patients [16]. The role of adjuvant treatments is still debated, although 5-fluorouracil and gemcitabine-based chemotherapy regimens are commonly adopted. Palliative chemotherapy represents the gold standard in the metastatic disease, mainly employing cisplatin and gemcitabine as first-line treatment in the pancreatobiliary subtype, and FOLFOX (oxaliplatin and 5-fluorouracil) in the intestinal one [17]. Despite the frequent utilization of next generation sequencing (NGS) in solid tumors, rare malignancies like ACs usually lack consensus-recognized molecular classifications and clinical studies supporting targeted treatment tailoring [1]. Finally, given the challenges posed by cancers arising in the pancreatobiliary anatomic district, a multidisciplinary approach is always critical to maximize AC patients survival [1, 3].

Here, we report a clinical case of a platinum-sensitive metastatic AC patient in which the identification of a *BRCA2* germline mutation drove a personalized multimodal approach with potential curative-intent.

## Case presentation

**Fig. 1:**
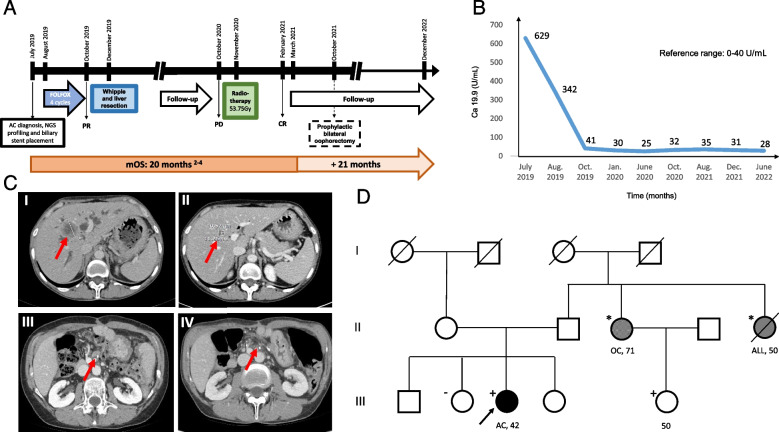
Schematic representation of patient’s clinical history. **A** Timeline summarizing main turning point of patient’s clinical history from initial diagnosis to last clinical and instrumental follow-up undergone in December 2022. **B** CA19.9 levels monitoring from diagnosis to last follow-up. **C** (I) Baseline CT scan performed in July 2019 and (II) assessment after 4 cycles of FOLFOX which demonstrated a major partial response in one of the liver lesions; (III) isolated retroperitoneal nodal relapse, nearby the superior mesenteric vessels, observed in October 2020 and (IV) the follow-up assessment at 36 months in which a complete response of nodal relapse was captured. The red arrows in the CT scans indicates the sites of neoplastic lesions (I, II, III) or their regression (IV). **D** Pedigree of the proband diagnosed with a germline BRCA2 W1692fs*3 mutation following its identification by somatic tumor next generation sequencing (NGS). The arrow indicates our patient (proband). Her sister, who tested negative for the same germline *BRCA2* mutation, is indicated by a (-) symbol. Instead, her female cousin resulted positive and is here indicated by a ( +) symbol. The number represents the age of *BRCA2* testing or cancer diagnosis, whichever comes first, and the organ represents the site of cancer origin (AC, ampullary cancer; OC, ovarian cancer; ALL, acute lymphoblastic leukemia). Roman numerals on the left edge represent generations. P, proband; squares, males; circles, females; oblique line, deceased; *, not tested; CR, complete response; PR, partial response; PD, progressive disease; NGS, Next Generation Sequencing; mOS, median overall survival

**Fig. 2:**
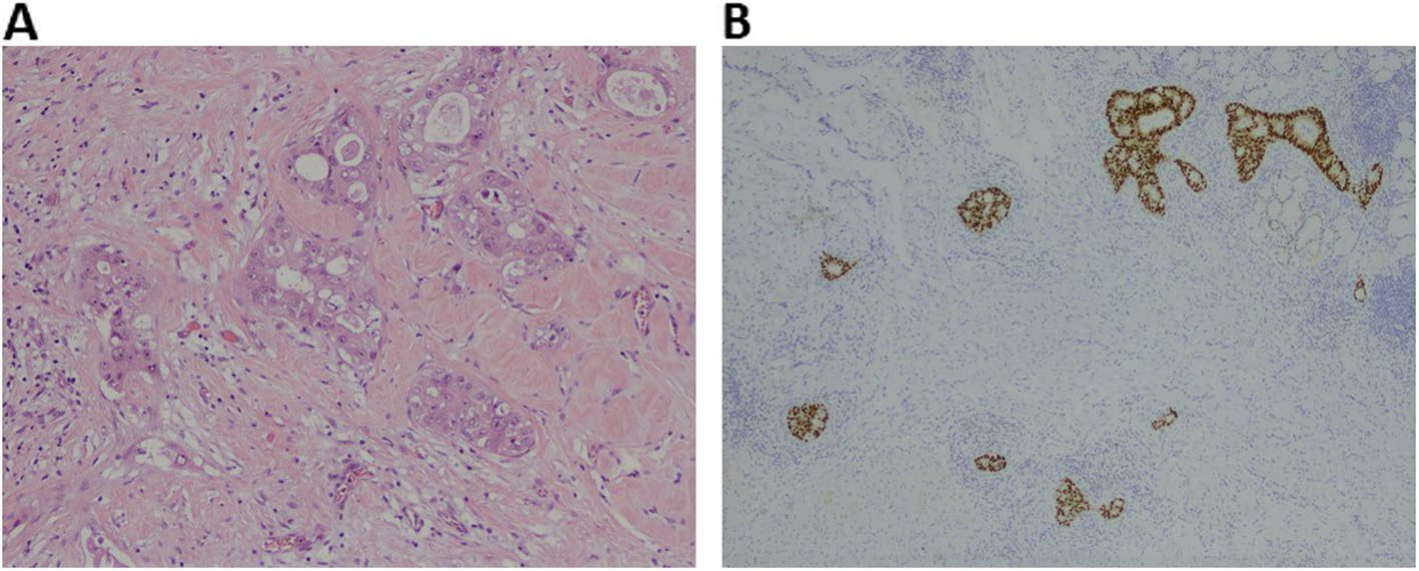
Representative images of patients’ tumor histology collected at surgical resection of the primary tumor. **A** Hematoxylin and eosin (H&E; × 40 view) stained section of ampullary adenocarcinoma. **B** Immunohistochemistry performed using a ready-to-use CDX2 antibody stained by DAKO-OMNIS (original magnification × 4)

In July 2019, a 42-year-old woman with unremarkable medical history referred to medical attention after the onset of recurrent abdominal pain. An ultrasound and CT scan revealed a tumor in the distal bile duct, associated with satellite pathological lymph nodes and three liver nodules measuring up to 37 mm in diameter (Fig. 1A). Based on the evidence of initial obstructive jaundice, an endoscopic retrograde cholangiopancreatography was performed for biliary stenting, with subsequent bilirubin normalization. Following, an endoscopic ultrasound-guided biopsy of the ampullary mass yielded a diagnosis of stage IV (metastatic), intestinal-type adenocarcinoma of the ampulla (Fig. 2). Baseline serum CA19.9 and CEA were 629 U/ml (reference range 0–35) and 11.9 ng/ml (reference range 0–5), respectively.

Next generation sequencing (NGS) of the tumor tissue was obtained through the FoundationOne CDx 324-genes panel. Among the alterations retrieved (Table 1), the NGS analysis revealed pathogenic mutations of *BRCA2* (W1692fs*3 with a variant allele frequency (VAF) of 44%) and *FANCA* (Q13FS*31 with 3% VAF).

**Table 1:** Next generation sequencing (NGS) performed on tumor tissue collected from primary tumor biopsy at the initial diagnosis of ampullary adenocarcinoma. The NGS analysis was performed by FoundationOne CDx within the GO40782 clinical trial. Variant allele frequency (VAF) data were made available only for *BRCA2* and *FANCA* genes upon clinical specific request to guide germline assessment in the context of patient’s genetic counseling

Gene	Alterations	VAF
***Genomic alterations identified***		
* BRCA2*	W1692fs*3	44%
* FANCA*	Q13fs*31	3%
* TP53*	F328fs*6	NA
* APC*	S1545*	NA
* NOTCH1*	W2034_A2035 > C	NA
* NOTCH1*	A2031fs*7	NA
***Variant of unknown significance identified***		
* TNFAIP3*	M112V	NA
* DOT1L*	F1474L	NA
* PIK3R1*	V718_A720del	NA
* ERCC4*	R477Q	NA
* DDR1*	E85_Q88del	NA
* KDM5A*	R1506Q	NA
* AR*	K809T	NA
* IDH2*	S10L	NA
***Advanced genomic analysis***		
* TMB*	11.35 mut/Mb	NE
* Microsatellite status*	NE	NE

*NA* Not available, *NE* Not evaluable, *TMB* Tumor mutational burden, *VAF* Variant allele frequency

After ruling out dihydropyrimidine dehydrogenase (DPYD) deficiency, the patient received a first cycle of FOLFOX treatment. Ahead of the second planned cycle, an afebrile grade (G) 4 neutropenia occurred. Following neutrophil normalization, the patient restarted the scheduled treatment receiving three further FOLFOX cycles with peg-filgrastim prophylaxis. However, ahead of the scheduled fifth cycle an afebrile G4 neutropenia and a concomitant G4 thrombocytopenia were documented. Given this enhanced hematological toxicity, the finding of somatic *FANCA* mutation, and the young age of the patient, Fanconi’s anemia was excluded by a negative diepoxybutane test.

A CT scan evaluation after 4 cycles of chemotherapy showed a major partial response (PR > 50%) with complete regression of two liver metastases, while the serum CA19.9 level dropped to 40.8 U/ml (Fig. 1B and C).

A genetic counseling was undertaken and the BRCA2 W1692fs*3 was confirmed germline; accordingly, the patient’s relatives were screened (Fig. 1D).

In light of the severe chemotherapy-induced myelotoxicity, the patient’s young age, technical resectability of the residual tumor burden, and the entity of the disease remission, radical resection of the primary tumor, locoregional lymph nodes, and liver sites of metastases was recommended by multidisciplinary team discussion. On December 4, 2019, the patient underwent a duodeno-cefalo-pancreatectomy and the anatomic resection of both liver segments II and VIII. The pathology report demonstrated a negative-margin (R0) resection of a ypT3b ypN1 ypM1 (liver) AC. A post-operative CT scan showed no evidence of disease (Fig. 1C).

In October 2020, after a 10-month follow-up period with regular CT scans and bio-humoral monitoring, an isolated retroperitoneal nodal tumor recurrence was detected nearby the superior mesenteric vessels. Thus, considering prior myelotoxicity and the expected enhanced sensitivity to radiotherapy in *BRCA2* mutant cancer, the patient underwent a long-course definitive imaging-guided radiotherapy (IGRT) for a total dose of 53.75 Gy in 25 fractions leading to a complete remission.

Following the identification of the *BRCA2* germline mutation and given the sustained absence of AC relapse, the patient went on a dedicated screening schedule, and in October 2021, she underwent prophylactic bilateral oophorectomy. As in December 2022, after 3 years from tumor surgical resection, the disease is still radiologically and biochemically undetectable (Fig. 1A, B, and C).

## Discussion and conclusions

*BRCA1/2* mutations are emerging as a potential therapeutic target of poly (ADP-ribose) polymerase (PARP) inhibitors (PARPi) in pancreatobiliary cancers, particularly in germline mutations carriers [14]. However, *BRCA* mutations can also predispose to sensitivity to standard DNA-damaging treatment options, including cytotoxic agents inducing double strand breaks such as oxaliplatin, or radiotherapy [18]. Accordingly, we reported a peculiar case in which the identification of a *BRCA2* germline mutation supported a multimodal personalized approach with curative intent for metastatic AC, for which surgical resection is not recommended [17].

In this case, we described how the detection of this hereditary gene alteration had several therapeutic implications beyond the potential opportunity of a targeted treatment with PARPi. First, *BRCA2* mutant cancers can be remarkably sensitive to platinum compounds, potentially predisposing to deep and sustained responses [18]. This encouraged us to take advantage of a profound response and consider a personalized surgical approach with potential curative intent both on primary and metastatic lesions. Second, germline *BRCA1/2* mutations have been associated, even though not univocally, with enhanced toxicity to platinum compounds [19–21]. This case is consistent with some of these previous reports, since we observed the occurrence of a G4 neutropenia despite granulocyte stimulating factor (G-CSF) support given as secondary prophylaxis. The impossibility of continuing FOLFOX together with the expected limited impact of a platinum-free second line treatment (i.e., FOLFIRI), reinforced the indication of a personalized surgical approach. Moreover, the patient’s young age and her good performance status had a critical role in driving the aggressiveness of our multimodal approach. Third, when nodal disease relapse later occurred, we reasoned that radiation therapy, based on the same BRCA-driven enhanced sensitivity to DNA damage, could have been the best treatment opportunity in terms of efficacy as well as safety [18]. Ultimately, we observed a complete response that is still ongoing (Fig. 1A). Fourth, as soon as we identified a *BRCA2* mutation through tumor somatic NGS, we recommended genetic counseling. This should be mandatory also in metastatic patients, and not only for their relatives given the chance to achieve a long survival, in order to access a dedicated screening program for risk reduction of further oncological diagnosis (Fig. 1D). Accordingly, after careful discussion of advantages and disadvantages related to the prognostic context of a metastatic disease, our patient decided to undergo prophylactic bilateral oophorectomy.

To better contextualize our report, we searched for other publications dealing with treatment outcomes in *BRCA2* germline mutant AC patients. Since ACs are usually analyzed in cohorts including also biliary and pancreatic cancers, we found only one other report specifically describing the clinical history of a *BRCA2* germline mutant stage III AC patient [22]. Differently from our report, the patient achieved only a minor radiological response to neoadjuvant gemcitabine plus cisplatin that was followed by surgical resection of the primary tumor. However, disease relapsed and the patient had only marginal benefit from other subsequent lines of treatment for advanced disease [22]. As reported in this case as well as learned from the POLO trial in pancreatic adenocarcinoma, harboring a *BRCA2* germline mutation does not directly confer platinum sensitivity to cancers [14]. Moreover, a potential different sensitivity to cisplatin and oxaliplatin cannot be excluded based on their different mechanisms of action [23]. We found no data dealing with PARPi efficacy in this rare subset of patients.

In conclusion, even considering the intrinsic limitations of a single clinical report, we suggest that the finding of *BRCA* germline mutations in ACs should be taken into consideration, together with other clinical variables, given their potential association with remarkable response to cytotoxic chemotherapy that might be burdened with enhanced toxicity. Indeed, *BRCA1/2* mutations might offer the opportunity of personalizing treatment beyond PARPi usage up to a multimodal approach with curative intent.

## Data Availability

Not applicable.
